# Local magnetic flux density measurements for temperature control of transient and non-homogeneous processing of steels

**DOI:** 10.1038/s41598-019-54503-5

**Published:** 2019-11-29

**Authors:** Gonçalo Sorger, Pedro Vilaça, Telmo G. Santos

**Affiliations:** 1 Department of Mechanical Engineering, School of Engineering, Aalto University, 02150 Espoo, Finland; 2 UNIDEMI, Department of Mechanical and Industrial Engineering, NOVA School of Science and Technology, Universidade NOVA de Lisboa, 2829-516 Caparica, Portugal

**Keywords:** Magnetic properties and materials, Ferromagnetism, Characterization and analytical techniques, Mechanical engineering

## Abstract

Measuring temperatures during high-temperature processing of steels is usually limited to surface measurements that cannot directly assess the internal temperature distribution. Here, we demonstrate the feasibility of using a magnetic flux density measurement system to assess transient and non-homogeneous temperature fields in a modern high-strength steel, within the intercritical temperature range where microstructural evolution defines their key mechanical properties. The system accurately detects the Curie temperature and distinguishes temperature change rates within the processed volume. The magnetic measurements are also sensitive to the volume above Curie temperature and its shape, as revealed when integrated with thermal computational simulations. The electromagnetic signal provides real-time qualitative and quantitative information relevant to the metallurgical conditions enabling future intelligent control systems for the production and processing of steels. Contactless measurements of temperature-dependent electromagnetic properties can enable through-thickness temperature monitoring solutions, opening up opportunities for non-destructive full-field imaging of steels during thermal and thermomechanical processing.

## Introduction

Today, temperature measurements during high-temperature processing of steels are limited to surface measurements and cannot directly assess the internal temperature distribution. Measurements of the electromagnetic properties are used in material characterization but mostly in isothermal and homogenous conditions. However, since the electromagnetic properties of steels are temperature-dependent they can be used to develop new solutions for contactless, non-destructive, temperature monitoring during high-temperature thermal and thermomechanical processing.

Steel is the most important engineering material used worldwide, hence any small increment in quality and energy efficiency during processing is significantly amplified, resulting in global benefits on economic, environmental and social impact. Nowadays, modern high-strength steels (HSS) offer superior mechanical properties, which enable leaner, lighter and, therefore, more cost-efficient applications. These steels are typically produced via thermomechanical controlled processing (TMCP)^1^, a production route that combines controlled rolling and fast cooling to generate the refined microstructure responsible for the high-strength alongside good toughness^2–4^. Controlling the temperatures during the final TMCP steps is crucial and the role of the intercritical temperature range, between A_1_ and A_3_, is particularly relevant^5–8^. Thermal and thermomechanical processing in this temperature domain promotes the microstructure transformation and grain refinement that are responsible for the enhanced mechanical properties^9,10^. Frequently, the application of these steels in the fabrication of structures and components requires some form of joining, typically welding. The sensitivity of the HSS to the high peak temperatures and cooling rates is well established for the widely used fusion welding methods^11,12^, including the destruction of the original microstructure and subsequent degradation of strength, toughness and fatigue life^13^. Solid-state thermomechanical welding processes, such as Friction Stir Welding (FSW), can overcome this limitation^14^. FSW of steel can be performed with peak temperatures inside the [A_1_, A_3_] range resulting in fine-grained microstructures with mechanical properties that match or even surpass those of the original base materials^15–17^. Reliable monitoring, both during production of the raw material and in any subsequent thermomechanical processing operation, can support intelligent control methods that will prevent the severe microstructure degradation occurring above the A_3_ temperature, and deliver better material properties^18–20^.

There are several methods for measuring temperatures but most are limited to laboratory applications and/or are not suited for high temperature industrial applications^21^. Typically, in industrial processes, temperatures are measured using thermocouples and pyrometers^22^ or infrared cameras^13,23^. The former are cumbersome since they require contact and only provide point measurements and the latter are expensive and quite dependent on surface conditions making it difficult to maintain calibration. Furthermore, both are limited to surface temperature measurements and cannot directly assess the internal temperature, which is critical when a significant in-depth temperature gradient exists or when the heat is generated internally, e.g. via plastic deformation or oxidation. Thus, there is a need for advanced temperature monitoring solutions, with a fast acquisition rate, enabling high-speed processing and through thickness assessment.

The critical temperatures associated with phase transformations can be identified indirectly with dilatometry measurements^24,25^. Differential scanning calorimetry (DSC) can also be used with the added benefit of detecting the ferromagnetic ↔ paramagnetic transformation at the Curie temperature, T_C_^26–28^. The electromagnetic properties of steel are directly related to microstructure. In isothermal conditions, electromagnetic measurements are used to study phase fraction^29–31^, hardness^32–34^, stress-states^35–37^, mechanical properties^38^, and surface treatments^39–41^. However, the temperature dependence of the electromagnetic properties^42,43^ can be further exploited to characterize and monitor the steels during transient thermal and thermomechanical processing^44–46^ with non-homogeneous conditions.

We developed a magnetic measurement system that monitors the local magnetic flux density variations, $\Delta |\overrightarrow{B}|$, caused by the change of relative magnetic permeability, μ_R_, with varying temperature inside the processed zone. The new measurement system was applied to a S700MC HSS to assess the thermal condition inside the through-thickness processed volume. Within the optimal processing temperatures for the HSS, [A_1_, A_3_], i.e. around T_C_, the signal is both qualitatively and quantitatively sensitive to temperature and to temperature change rates. The concept behind this new system was initially conceived for the control of FSW^47^. The goal was to combine the benefits of the solid-state thermomechanical FSW process with a real-time monitoring of the full field processing temperatures. Soon it became clear that this concept is applicable to a wide range of other relevant steel processing methods (with the appropriate sensor architecture development for each specific application). Thus, this scientific communication is presented in a generalized approach to thermal and thermomechanical processing applications were it is beneficial that the peak temperatures are inside, or in the vicinity, of the intercritical temperature region.

## Results

### Experimental setup

**Figure 1:**
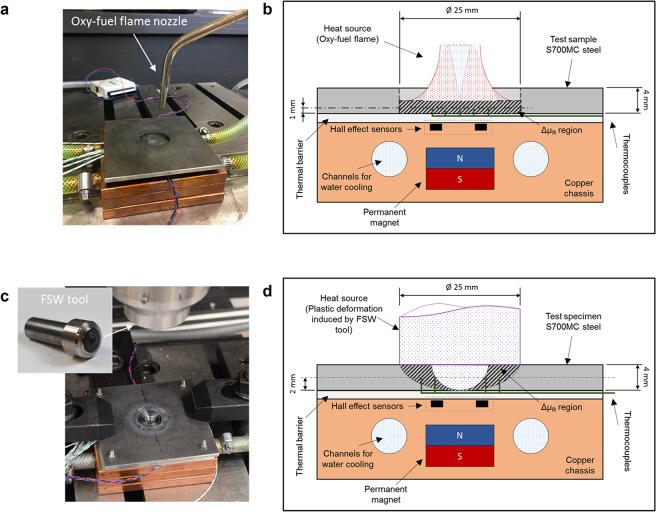
Schematic representation of the experimental setup: (**a**) Photo of the experimental setup for the thermal cycle using the oxy-fuel flame as the heat source; (**b**) Photo of the experimental setup for the thermomechanical cycle using the plastic deformation induced by the FSW tool as the heat source; (**c**) Details of the measurement system inside the chassis shown for the thermal cycle; and (**d**) Details of the measurement system inside the chassis shown for the thermomechanical cycle.

We developed a magnetic measurement system tailored to identify temperature evolution in the ideal range for thermomechanical processing of HSS. Figure 1 shows schematic representations of the measurement setup created to monitor the magnetic flux density during thermal and thermomechanical cycles. The setup consists of a S700MC HSS test specimen clamped to a non-magnetic copper chassis (Fig. 1a) containing the measurement system. Inside the chassis, a permanent magnet generates a magnetic field, $\overrightarrow{H}$, that interacts with the steel specimen, changing the magnetic flux density, $\overrightarrow{B}$, according to the relation $\overrightarrow{B}={\mu }_{R}\cdot \overrightarrow{H}$, assuming that magnetic permeability, *μ*_*R*_, is a scalar since the material is considered isotropic. Two (for redundancy) Hall-effect sensors, positioned between the magnet and the steel, monitor the local magnetic flux density. The location of these sensors was chosen based on the results of the computational analysis presented in the ‘Computational analysis’ section. Thermocouples positioned at the half-thickness of the processed zones, according to Fig. 1b, measured the temperatures. The chassis was water cooled to protect the magnet and the magnetic sensors from the effects of the high temperatures. An oxy-fuel flame was used as the heat source for the thermal cycles (Fig. 1c), and plastic deformation induced by a rigid friction stir welding (FSW)^48,49^ tool was the heat source for the thermomechanical cycles (Fig. 1d). These two heat sources were selected to decouple the purely thermal effect from any eventual effect of the material flow during the thermomechanical processing that might affect the magnetic measurements. Non-electrical heat sources were selected to avoid any coercive electromagnetic interaction with the magnetic measurement system. A more detailed description of the experimental setup and procedure is described in the ‘Methods’ section and in the Supplementary Information file accompanying the online version of this publication.

### Phase transformation and Curie temperatures of S700MC steel

**Figure 2:**
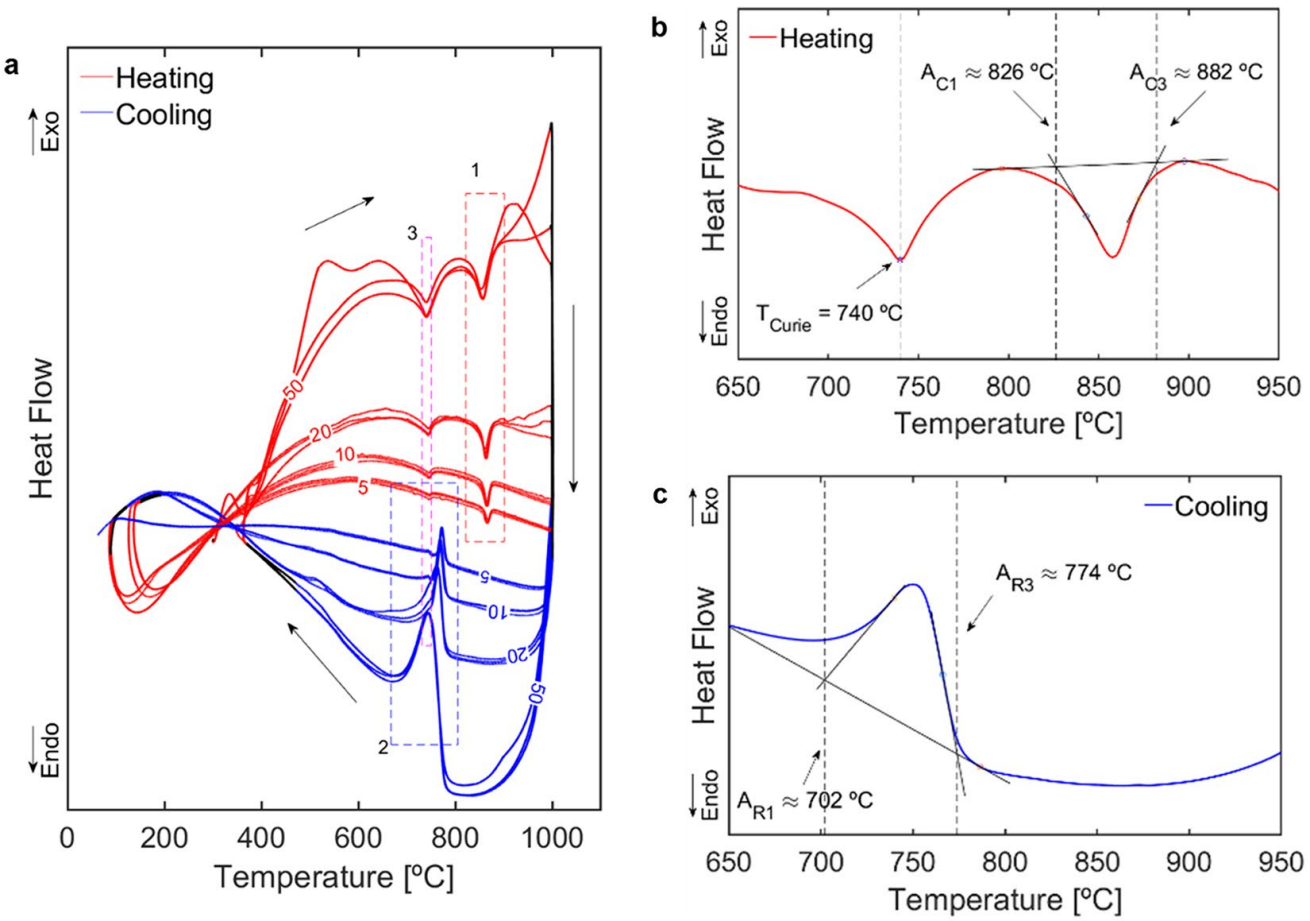
Heat flow vs temperature measured via differential scanning calorimetry (DSC). Red lines correspond to heating while blue lines correspond to cooling: (**a**) Full set of twelve thermal cycles labelled 5, 10, 20 or 50 according to heating/cooling rate [°C/min]. Dashed rectangles labelled 1 and 2 mark the intercritical temperature regions. Dashed rectangle labelled 3 marks the magnetic transformation peaks; (**b**) Detail of 50 °C/min heating with T_C_ ≈ 740 °C, A_C1_ ≈ 826 °C and A_C3_ ≈ 882 °C; (**c**) Detail of 50 °C/min cooling with A_R3_ ≈ 774 °C and A_R1_ ≈ 702 °C.

DSC measurements with different heating and cooling rates (detailed in the ‘Methods’ section) revealed the presence of three distinct peaks in each cycle. These are labelled 1, 2 and 3 in Fig. 2a. The endothermic peaks inside 1 identify the body centered cubic α iron (e.g. ferrite) → face centred cubic γ iron (austenite) phase transformation between austenite onset temperature, A_C1_, and austenite finish temperature, A_C3_, in the heating stage. The exothermic peaks inside 2 mark the γ → α transformation region between the ferrite onset temperature, A_R3_, and ferrite finish temperature, A_R1_, in the cooling stage. The peaks inside 3 identify T_C_ and their magnitude is smaller than the phase transformation peaks. As with other steels^27^, the phase change temperatures depend on the heating/cooling rate but T_C_ is quite stable.

A closer analysis of the endothermal reactions during heating at 50 °C/min is presented in Fig. 2b. The peak between 700 and 800 °C corresponds to the ferromagnetic to paramagnetic transformation and its lowest point defines T_C_ = 740 °C. The peak between 800 and 900 °C marks the α ↔ γ phase transformation. We extrapolated the A_C1_ and A_C3_ temperatures by tracing the baseline of the peak and intersecting it with the tangents to the inflection points of the curves. The temperature where the negative slope tangent intersects the baseline defines A_C1_ = 826 °C and the temperature where the positive slope tangent intersects the baseline defines A_C3_ = 882 °C. The austenite to ferrite transformation onset and finish temperatures on cooling, A_R3_ and A_R1_, were determined following the same methodology (Fig. 2c). Since for the 50 °C/min cooling, the T_C_ is inside the [A_R1_, A_R3_] temperature range, the magnetic transformation occurs within the phase transformation and the energetic contributions from these two phenomena are added forming a single exothermic peak. Considering the relation of the T_C_ with the equilibrium transformation temperatures [A_1_, A_3_], an acceptable approximation is [A_1_, A_3_] ≈ [A_R1_, A_C3_].

### Computational analysis

**Figure 3:**
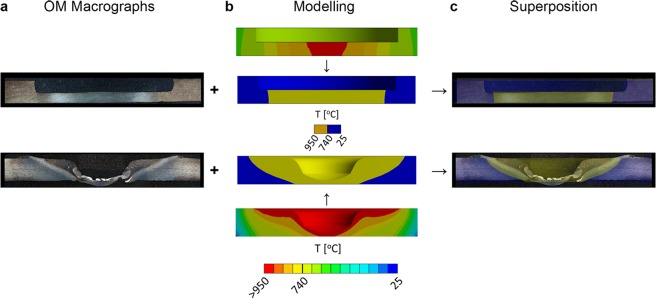
Thermal modelling analysis and macrographs obtained experimentally. The images on the top and bottom half of the figure correspond to the thermal and thermomechanical processing conditions, respectively. **(a**) Optical macrographs showing the heat-affected zones (OM Macrographs). (**b**) Full temperature field map and map of the regions with T < T_C_ and μ_R_ ≫ 1 (blue) and T > T_C_ and μ_R_ ≈ 1, V_paramagnetic_, (yellow) obtained via computational thermal analysis (Modelling). (**c**) Superposition of the images reveals a good agreement between the experimental and computational results.

To estimate the temperature distribution for the experimental part of this work and to support the design of the magnetic measurement system, we performed computational thermal analyses using the Finite Element Method (FEM). The analysis established the full temperature fields and identified the volume of material that reaches temperatures above T_C_, thus, changing from μ_R_≫ 1 to μ_R_ ≈ 1 and becoming paramagnetic, hereafter the paramagnetic volume, V_paramagnetic_ (Fig. 3). The conditions for the steady-state thermal analysis are described in the ‘Methods’ section. Superimposing the modelled V_paramagnetic_ on the etched cross section images of the test specimens reinforces the good agreement between the V_paramagnetic_ obtained by computational analysis and the thermomechanically-affected zones (TMAZ) obtained experimentally, further confirming the validity of the model.

**Figure 4:**
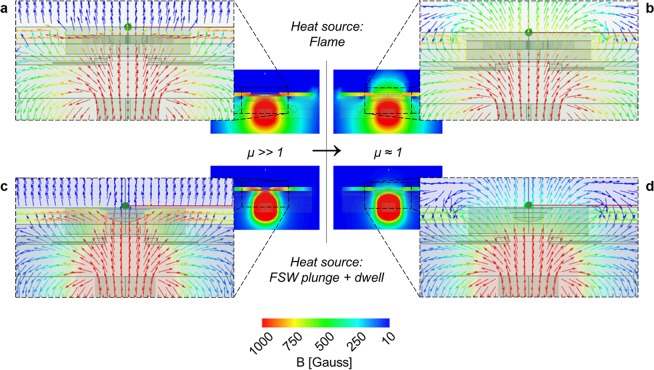
Magnetic flux density maps for the thermal processing with oxy-fuel (**a,b**) and for the thermomechanical processing with FSW tool (**c,d**). The computational analysis reveals the difference in density and direction of the magnetic flux with μ_R_Vparamagnetic_ ≫ 1, when T_Vparamagnetic_ < T_C_, depicted in (**a,c**), compared to when μ_R_Vparamagnetic_ ≈ 1, when T_Vparamagnetic_ > T_C_, depicted in (**b,d**). The small black rectangles represent the positions of the probes.

In a multi-physical approach, we then integrated V_paramagnetic_ into a magnetostatic computational analysis to study the interaction of the magnetic field with the test plate upon the ferromagnetic to paramagnetic transformation. The results provide a visualization of the difference between the magnetic flux density in the presence of a V_paramagnetic_ in the fully ferromagnetic condition: μ_R_ ≫ 1 when T < T_C_ (Fig. 4a,c); *versus* V_paramagnetic_ in the paramagnetic condition: μ_R_ ≈ 1 when T > T_C_ (Fig. 4b,d). These results were used to support the design of the magnetic measurement system. The optimal position for the sensors is where the magnetic flux density experiences the greatest variation, comparing the ferromagnetic to paramagnetic states. This occurs mainly inside the paramagnetic volume itself and in its immediate vicinity. Since the inside of the material is inaccessible and the heat sources occupy the space above the V_paramagnetic_, the magnetic sensors were positioned below V_paramagnetic_.

### Experimental measurements of temperature and magnetic permeability during thermal and thermomechanical cycles

**Figure 5:**
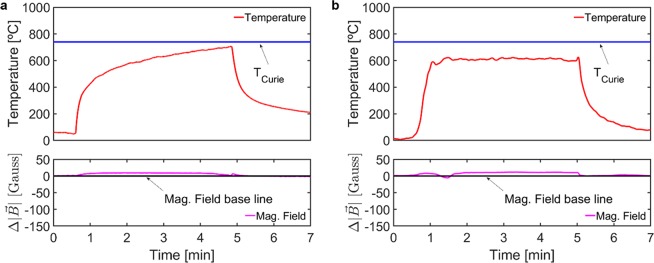
Temperature and magnetic flux density measurements for the cycles with T_MAX_ < T_C_ using (**a**) oxy-fuel flame and (**b**) FSW plunge and dwell as heat source.

**Figure 6:**
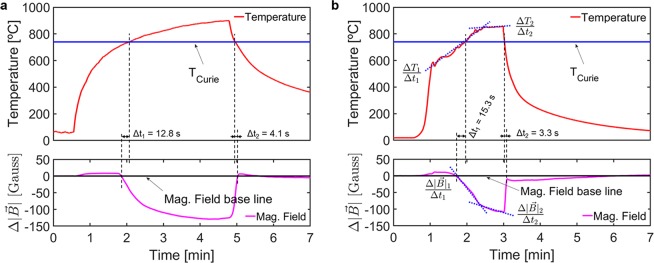
Temperature and magnetic flux density measurements for the cycles with T_MAX_ > T_C_ using (**a**) oxy-fuel flame and (**b**) FSW plunge and dwell as heat source.

The magnetic measurement system, introduced in the ‘Experimental setup’ section, was used to monitor the magnetic flux density and the temperature during the thermal and thermomechanical cycles (see details in ‘Methods’ section). The different cycles are identified according to heat source - Flame or FSW tool - and maximum temperature above *or* below the Curie temperature: T_MAX_ > T_C_ *or* T_MAX_ < T_C_. In Figs. 5 and 6, the plotted temperature curves correspond to the thermocouple that registered the highest temperature in each measurement (i.e. the one closest to the heat source). The magnetic measurements are represented as magnetic flux density variation, $\Delta |\overrightarrow{B}|$, from a baseline defined as the initial value of $|\overrightarrow{B}|$. Positive values of $\Delta |\overrightarrow{B}|$ indicate increases of μ_R_ in the processed material whereas the opposite applies for negative values of $\Delta |\overrightarrow{B}|$.

The results for the Flame_T_MAX_ < T_C_ cycle are presented in Fig. 5a. The maximum temperature does not go over T_C_ and there is no drop in magnetic flux density, indicating that T_C_ was not reached anywhere in the specimen. Similarly, in the FSW_T_MAX_ < T_C_ cycle (Fig. 5b) the maximum temperature measured did not exceed T_C_ and no drop in Δ$|\overrightarrow{B}|$ occurs. It is worth mentioning that even though the whole processed volume of the steel specimens is kept below the Curie temperature the Δ$|\overrightarrow{B}|$ moves slightly away from the baseline. This phenomena of sensitivity of the magnetic measurement to different temperatures below the T_C_ is reported by other authors^50–52^.

The Flame_T_MAX_ > T_C_ measurements are shown in Fig. 6a. T_MAX_ surpassed T_C_ and, accordingly, the magnetic flux sensors detected a clear decrease in signal strength, followed by a return to the baseline when the temperature dropped below T_C_ at the end of the cycle. The magnetic sensors registered the signal decrease about Δt_1_ = 12.8 seconds before the thermocouple recorded values above T_C_. This offset is due to the fact that the thermocouple provides a point measurement while the magnetic measurement is immediately sensitive to the change in μ_R_, caused by temperatures above T_C_, anywhere in the volume of processed material, starting from the vicinity of the power source. This delay is opposite during the cooling, for the same reason. Note that Δt_2_ <  Δt_1_ which reflects the difference in temperature change rate between heating and cooling periods.

The FSW_T_MAX_ > T_C_ measurements, presented in Fig. 6b, exhibit all the same characteristics as those described for Flame_T_MAX_ > T_C_, and contain additional distinguishable features of the notable significance. The distinguishable effect, depicted in Fig. 6b, is an inverse systematic relation between the $\Delta |\overrightarrow{B}|$ rate and the heating rate. During the dwell phase of the thermomechanical cycle, the temperature curve exhibits two different heating rates, represented by blue dotted lines marked $\frac{\Delta {T}_{1}}{\Delta {t}_{1}}=3.4$ and $\frac{\Delta {T}_{2}}{\Delta {t}_{2}}=0.2$ °C/s. The two heating rates are reflected in the $\Delta |\overrightarrow{B}|$ *versus* time graph as indicated by blue dotted lines marked $\frac{\Delta {|\overrightarrow{B}|}_{1}}{\Delta {t}_{1}}=-\,1.1$ and $\frac{\Delta {|\overrightarrow{B}|}_{2}}{\Delta {t}_{2}}=-\,0.7$ Gauss/s. This means that the measurement system not only accurately detects the T_C_, but is also sensitive to the effect of temperature change rates within the processed volume. This quantitative effect is of the upmost importance because it has not been found in the existing literature and it opens up the possibility of implementing new intelligent control systems. As an example, the Δ$|\overrightarrow{B}|$ signal can be used as an input for a control system to maintain the temperatures during steel production or processing inside the [≈A_1_,T_C_] range. The results of a more detailed investigation of the sensitivity of the $\Delta |\overrightarrow{B}|$ to the volume above Curie temperature, V_paramagnetic_, will be presented next.

### Magnetic flux density measurements versus material volume above Curie temperature

**Figure 7:**
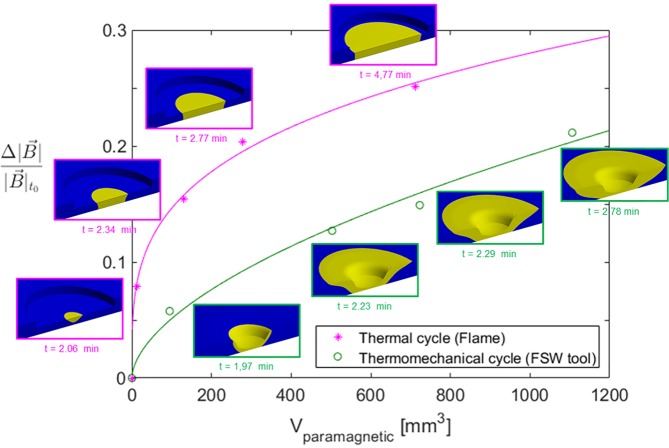
Relative magnetic flux density variation $(\frac{\Delta |\overrightarrow{B}|}{{|\overrightarrow{B}|}_{{t}_{0}}})$ *versus* paramagnetic volume $\,({V}_{Paramagnetic})$, during the heating period of the thermal cycle (Flame) and thermomechanical cycle (FSW tool). The data was fitted via power law curves, $\frac{\Delta |\overrightarrow{B}|}{{|\overrightarrow{B}|}_{{t}_{0}}}=k\cdot {{V}_{Paramagnetic}}^{n}$, where for the thermal cycle data: $k=0.03993$; $n=0.282$; and for the thermomechanical cycle data: $k=0.003636$; $n=0.5744$.

We estimated V_paramagnetic_, i.e. the volume of material with T > T_C_, at five different instants during the heating phase of Flame_T_MAX_ > T_C_ and FSW_T_MAX_ > T_C_ via computational analysis. Figure 7 shows the relationship between V_paramagnetic_ and the change in magnetic flux density $\varDelta |\overrightarrow{B}|$ relative to the initial value ${|\overrightarrow{B}|}_{{t}_{0}}$. Visual representations of the different volumes are included to support the interpretation of results. Power law curves, *f*(*x*) = *kx*_*n*_, where $x={V}_{paramagnetic}$ and $f(x)=\frac{\Delta |\overrightarrow{B}|}{{|\overrightarrow{B}|}_{{t}_{0}}}$, were fitted to the thermal cycle data ($k=0.03993$; $n=0.282$) and to the thermomechanical cycle data ($k=0.003636$; $n=0.574$). The two curves show that though ΔV_paramagnetic_ is smaller in the thermal cycle (Flame) than in the thermomechanical cycle (FSW tool) it has a stronger effect on $\frac{\varDelta |\overrightarrow{B}|}{{|\overrightarrow{B}|}_{{t}_{0}}}$.

Observing the visual representations of the paramagnetic volumes we can identify two different stages in the evolution of ${V}_{paramagneti{c}_{}}$. The first stage is dominated by an increase in the thickness of ${V}_{paramagneti{c}_{}}$ (i.e. $\varDelta V\propto \varDelta t$, where t is the thickness) and is more pronounced in ${{\rm{V}}}_{paramagneti{c}_{(Flame)}}$. The second stage is dominated by radial expansion (i.e. $\varDelta V\propto \varDelta d$, where *d* is the diameter) and is more evident in the evolution of ${{\rm{V}}}_{paramagneti{c}_{(FSWtool)}}$. These results suggest that $\frac{\varDelta |\overrightarrow{B}|}{{|\overrightarrow{B}|}_{{t}_{0}}}$ is sensitive to the shape of the paramagnetic volumes and to the way those shapes evolve over time.

## Discussion

The peak temperatures and the temperature change rates control the grain size evolution, precipitation and solution, which along with the strain hardening are responsible for the microstructure of steels and their mechanical properties. These phenomena depend mostly on the thermal and thermomechanical history across the intercritical temperature domain^12^, where the Curie temperature lies and the α ↔ γ phase transformation of iron structures occurs.

We developed a non-destructive, contactless, magnetic-based measurement system that exploits the temperature dependence of the electromagnetic properties to assess temperature domains inside a volume of processed steel. By monitoring the change of magnetic flux density near the Curie temperature, our system provides real-time qualitative and quantitative information related to the temperature field within the intercritical temperature domain. This enables the development of intelligent systems capable of assessing and controlling the phenomena that govern the metallurgical and mechanical properties of steels, which is of special relevance in the thermal and thermomechanical processing of modern high-strength steels. Examples of processes associated with an external heat source resulting in thermal processing, approximated in the paper by the thermal cycles using the flame as external the heat source, are i) with fusion: oxi-fuel welding and cutting, laser welding and cutting, electron beam welding, electric arc based welding and cutting; ii) without fusion: heat treatment, e.g. plasma, flame, high-frequency induction (where the sensor can be integrated with the induction coil). Examples of processes associated with thermomechanical processing, where the heat source is internal, i.e. the material, by energy dissipation during its bulk plastic deformation, are the FSW (partially addressed in the paper by the thermomechanical cycles using the plunge and dwell stages of a FSW tool), the friction welding, forging, hot rolling during steel production.

The multiphysical research plan encompassed DSC experimental measurements, computational thermal analysis and dedicated design and implementation of the magnetic-based measurement system. The DSC measurements with different heating and cooling rates were used to establish, with precision and reliability, the Curie temperature, T_C_ ≈ 740 °C, and the transformation temperatures, [A_R1_, A_C3_] = [702 °C, 882 °C], of the S700MC high-strength steel used as test specimen. The transformation temperatures considered were the ones obtained with the 50 °C/min rate as they are the ones closer to real processing conditions. The Curie temperature showed no dependence on the heating or cooling rates. The computational thermal analysis was used to support the design of the magnetic measurement system and identify the volume of material that reaches temperatures above T_C_, thus, changing from μ_R_ ≫ 1 to μ_R_ ≈ 1 and becoming paramagnetic.

The results show that our magnetic flux density measurements can be used to detect the magnetic transformation of the processed volume when it reaches the Curie temperature. Above this temperature, it is also sensitive to the effect of temperature change rates within the processed volume. Additionally, based on the multiphysical approach, the measurements provide sensitive data related to the evolution of the processed volume, namely, it can discern between different heating and cooling rates and between the effects of different changes in shape (Δ thickness vs Δ diameter). There is a $\varDelta |\overrightarrow{B}|$ associated with a small magnetic permeability increase below the Curie temperature indicating that the temperatures inside processed volume are changing, this may also represent a valuable information for a control system and should be studied in more detail.

The new and distinguishable results presented in this work result from two major differences in the implemented research plan when compared with the state of the art in this field, namely: (i) the measurements of the magnetic properties are performed during transient thermal processing with complex non-homogenous conditions; (ii) besides an external purely heat power source (i.e. a flame), the work included thermomechanical processing. In this case, the specimen materials become the heat power source, due to the heat generated by internal viscous energy dissipation during the non-homogeneous plastic deformation of the material and superficial frictional energy dissipation in the contact with the rigid FSW tool. The internal energy dissipation decreases as the temperature increases and, so, the thermomechanical processing resulted in steady-state heating rates, different for different stages of the deformation. This yielded the opportunity for the magnetic measurement system to show unique capabilities, besides accurately detecting the T_C_, such as being sensitive to the effect of temperature change rates within the processed volume. For full temperature range measurements other complementary measurement methods, such as pyrometers and infrared thermography systems, can be integrated via data fusion processes.

This through-thickness, contactless, monitoring solution (that can provide real-time data on the temperatures and temperature rates inside a volume of material under non-uniform and transient conditions) will contribute to bring steel production and processing in line with the new digitalization paradigm by providing large amounts of information rich data that can be readily available to support highly integrated, smart, cyber-physical manufacturing systems^53^. Going digital in the fabrication of steel-based structural systems and products, will allow zero-defect factories with safer working conditions and lower environmental impact.

The next steps will be to expand the capabilities of the measurement system from a single Hall-effect sensor configuration to multiple sensors forming an array. This will provide data with spatial resolution and enable image reconstruction of the processed volume of material. With a dedicated multiphysical solution, integrating computational simulation of the metallurgical evolution and thermal analysis with the non-destructive real-time magnetic measurement system presented here, it will be possible to deliver a full field imaging solution for application in intelligent automated control systems for steel production and processing.

## Methods

### Material

The base material used in this study was a S700MC high-strength steel produced by TMCP, whose chemical composition is: [max. wt.%] 0.059 C; 0.205 SI; 1.79 Mn; 0.007 P; 0.002 S; 0.026 Al; 0.083 Nb; V; 0.013; 0.113 T. The specimens used in the thermal and thermomechanical processing cycles were 100 × 100 × 4 mm plates. For the thermal cycles (oxy-fuel flame heat source), a region of reduced thickness (2 mm) was produced on the middle of the plates, by removing Ø 25 × 2 mm of material by machining as depicted in Fig. 1c, to concentrate the heating effect. Similarly, for the thermomechanical cycles (FSW tool heat source) a Ø 10 mm and 3.5 mm deep hole (Fig. 1d) was drilled at the center of the plates to remove a volume of material roughly equivalent to that of the tool probe.

### Differential scanning calorimetry (DSC)

Differential scanning calorimetry measurements were performed on a NETZSCH STA 449F1 equipment, capable of a maximum heating rate of 50 °C/s. A sample of the S700MC steel (approximately 1.5 × 2 × 3 mm and 75 mg) was placed inside an Al_2_O_3_ ladle and the measurements were carried out under a protective Argon atmosphere. The thermal cycles comprised four stages: 1 - holding at 100 °C; 2 – heating (at 50, 20, 10, and 5 °C/min); 3 - holding at 1000 °C; and 4 – cooling (at 50, 20, 10, and 5 °C/min). The cycles were carried out three times per heating/cooling rate. The holding times were 5 min.

### Computational analyses

Transient thermal computational analyses were performed using the commercial ANSYS Workbench 19.0 software. The purpose of these analyses was to obtain the temperature fields in agreement with the temperature measurements obtained experimentally via thermocouples and to estimate the volume of material that reached temperatures above T_C_. The geometries used were the same as shown in Fig. 1a and 1c, with the specific sample geometry for each heat source (i.e. a region of reduced thickness in the case of the flame as heat source, and the negative of the FSW tool in the case of the FSW tool as the heat source). The meshes were comprised of about 3.8 million (flame as heat source case) and 3 million (FSW tool as heat source case) tetrahedron elements. The maximum element size was 0.5 mm in the steel plates and 1 mm in all other bodies. The loading condition representing the flame was a heat flow with a normal distribution applied on the surface of the reduced thickness region of the steel specimen body. The analysis was carried out in one step. The loading conditions representing the effect of the heat flow from the FSW tool were applied on the surfaces corresponding to the negative of the tool geometry. The analysis was carried out in two steps. The first step with heat flow applied only on the surfaces corresponding to the probe, and the second step adding the heat flow contribution on the surfaces corresponding to the shoulder. The final results were obtained by adjusting loading conditions iteratively until the simulated temperature fields were in close agreement with the thermocouple measurements for each case. In both cases, the initial temperature was 25 °C, and a constant temperature of 25 °C was applied to the surfaces corresponding to the inside of the copper cooling tubes. An emissivity of 0.3 was considered at the top surface of the steel specimen, excluding the heat flow loading surfaces. All other outside surfaces were adiabatic. The material models used were those for Steel 1010, Copper, Titanium, and Air, available in the materials library of the ANSYS Workbench 19.0 software. The results were validated by comparing the temperature fields above the T_C_, obtained computationally, with the heat-affected zones evaluated in cross-sections of samples extracted from the center of the processed specimens. A table with the material thermal properties, figures and graphs supporting the methods implemented in the thermal analyses are included in the “Suplementary Information” file available with the online version of this paper.

The magnetostatic computational analyses were performed using the ANSYS Maxwell R18.0 software. The geometries were the same as in the thermal analyses. The meshes were comprised of 2.8 million tetrahedron elements (in both the flame and the FSW tool analyses). The volumes obtained from the thermal analysis were integrated into the magnetostatic model and the analyses were carried out for different values of magnetic permeability (μ_R_ ≫ 1 vs μ_R_ ≈ 1) in those volumes. The material models used were those for Steel 1010, Copper, Titanium, Air and NdFe35, available in the materials library of the ANSYS Maxwell R18.0 software. A table with the magnetic properties considered for the materials is included in the “Suplementary Information” file available with the online version of this paper.

### Thermal and thermomechanical cycles with temperature and magnetic flux density measurements

Thermal and thermomechanical cycles were carried out on S700MC HSS using non-electrical heat sources to avoid any coercive electromagnetic interaction with the magnetic measurement system. Two heat sources were applied to the specimens: For the thermal cycles it was an oxy-fuel flame, which is a purely thermal heat source; and for the thermomechanical cycles it was the thermomechanical processing, which is an indirect heat source via internal friction dissipation during the plastic deformation induced in the HSS specimen by the plunging and rotation of a rigid FSW tool. The tool material was a non-magnetic polycrystalline cubic boron nitride in a tungsten rhenium metal matrix. Thermocouples were inserted into small holes reaching the half thickness of the processed zone of the specimens. A spacing of 5 mm was kept between each thermocouple as shown in Supplementary Figure S2 in the Supplementary Information file.

The ‘Experimental setup’ section described the main features of test setup, depicted in Fig. 1. The magnet used to generate the magnetic field was a Ø 15 × 8 mm NeFeB permanent magnet with N42 magnetization in the axial direction. The distance from the magnet to the test sample was such that the interaction between the field and the sample generates the largest field intensity variation when the material changes from ferromagnetic to paramagnetic (and vice versa) without saturating the signal from the Hall-effect sensors. A good compromise between these two conditions was achieved at a distance of 13 mm, directly under the processed zone. The two sensors used to measure the magnetic flux density were SS496A1 ratiometric Hall-effect sensors. These were positioned in 2 mm deep slots machined on the copper chassis at ±5 mm from the center of the plate. The positioning of these sensors, relative to the permanent magnet and the test plate, was supported by the results of the magnetostatic computational analysis. The chassis was water cooled to protect the magnet and the magnetic sensors from the effects of the high temperatures. Furthermore, a 3 mm thick thermal barrier (air gap for the thermal cycles and a Ti plate for the thermomechanical cycles) was placed between the copper chassis and the steel test piece for additional protection of the magnet and the sensors from the high temperature, and also to provide additional backing support in the case of thermomechanical cycles. The data from the Hall-effect sensors and the thermocouples was acquired via a NI USB-6008 module and a NI-9212 module, respectively. A custom-made application was created in LabVIEW to control and synchronize the data acquisition and recording. The data acquisition rate used was 10 Samples/s (10 Hz) which was sufficient to capture with high resolution all the magnetic flux density gradients in the tested thermal and thermomechanical transient processes.

## Supplementary information

Supplementary Information

## Data Availability

Supplementary information is available in the document accompanying the online version of this publication.

## References

[1] Zhao J, Jiang Z (2018). Thermomechanical processing of advanced high strength steels. Prog. Mater. Sci..

[2] Zhao MC, Yang K, Shan Y (2002). The effects of thermo-mechanical control process on microstructures and mechanical properties of a commercial pipeline steel. Mater. Sci. Eng. A.

[3] Xue XH, Shan YY, Zheng L, Lou SN (2006). Microstructural characteristic of low carbon microalloyed steels produced by thermo-mechanical controlled process. Mater. Sci. Eng. A.

[4] Endo S, Nakata N (2015). Development of Thermo-Mechanical Control Process (TMCP) and high performance steel in JFE Steel. JFE Tech. Rep..

[5] Movahed P, Kolahgar S, Marashi SPH, Pouranvari M, Parvin N (2009). The effect of intercritical heat treatment temperature on the tensile properties and work hardening behavior of ferrite-martensite dual phase steel sheets. Mater. Sci. Eng. A.

[6] Shi L (2014). Improved toughness and ductility in ferrite/acicular ferrite dual-phase steel through intercritical heat treatment. Mater. Sci. Eng. A.

[7] Shukla R, Ghosh SK, Chakrabarti D, Chatterjee S (2013). Microstructure, texture, property relationship in thermo-mechanically processed ultra-low carbon microalloyed steel for pipeline application. Mater. Sci. Eng. A.

[8] Cao W (2017). Ultrahigh Charpy impact toughness (<450J) achieved in high strength ferrite/martensite laminated steels. Sci. Rep..

[9] Humphreys FJ, Prangnell PB, Priestner R (2001). Fine-grained alloys by thermomechanical processing. Curr. Opin. Solid State Mater. Sci..

[10] Arora HS (2019). High Tensile Ductility and Strength in Dual-phase Bimodal Steel through Stationary Friction Stir Processing. Sci. Rep..

[11] Kim BC, Lee S, Kim NJ, Lee DY (1991). Microstructure and local brittle zone phenomena in high-strength low-alloy steel welds. Metall. Trans. A.

[12] Górka J (2018). Assessment of Steel Subjected to the Thermomechanical Control Process with Respect to Weldability. Metals (Basel)..

[13] Górka J, Janicki D, Fidali M, Jamrozik W (2017). Thermographic Assessment of the HAZ Properties and Structure of Thermomechanically Treated Steel. Int. J. Thermophys..

[14] Thomas WM, Threadgill PL, Nicholas ED (1999). Feasibility of friction stir welding steel. Sci. Technol. Weld. Join..

[15] Lienert T, Jr WS (2003). Friction stir welding studies on mild steel. Weld. J. Res. Suppl..

[16] Morisada Y (2013). Improvement of toughness and strength of thick structural steel weld by friction stir welding conditions. Sci. Technol. Weld. Join..

[17] Xue P, Komizo Y, Ueji R, Fujii H (2014). Enhanced mechanical properties in friction stir welded low alloy steel joints via structure refining. Mater. Sci. Eng. A.

[18] Fujii H (2006). Friction stir welding of carbon steels. Mater. Sci. Eng. A.

[19] Sorger Gonçalo, Lehtimäki Eero, Hurme Susanna, Remes Heikki, Vilaça Pedro, Molter Lars (2017). Microstructure and fatigue properties of friction stir welded high-strength steel plates. Science and Technology of Welding and Joining.

[20] Sorger Gonçalo, Sarikka Teemu, Vilaça Pedro, Santos Telmo G. (2018). Effect of processing temperatures on the properties of a high-strength steel welded by FSW. Welding in the World.

[21] Childs PRN, Greenwood JR, Long CA (2000). Review of temperature measurement. Rev. Sci. Instrum..

[22] Müller B, Renz U, Hoppe S, Klocke F (2004). Radiation Thermometry at a High-Speed Turning Process. J. Manuf. Sci. Eng..

[23] Davies MA, Ueda T, M’Saoubi R, Mullany B, Cooke AL (2007). On The Measurement of Temperature in Material Removal Processes. CIRP Ann. - Manuf. Technol..

[24] Kop TA, Sietsma J, Van Der Zwaag S (2001). Dilatometric analysis of phase transformations in hypo-eutectoid steels. J. Mater. Sci..

[25] García De Andrés C, Caballero FG, Capdevila C, Álvarez LF (2002). Application of dilatometric analysis to the study of solid-solid phase transformations in steels. Mater. Charact..

[26] Kargul T (2017). Investigations of Temperatures of Phase Transformations of Low-Alloyed Reinforcing Steel within the Heat Treatment Temperature Range. Arch. Metall. Mater..

[27] Raju S, Ganesh BJ, Banerjee A, Mohandas E (2007). Characterisation of thermal stability and phase transformation energetics in tempered 9Cr-1Mo steel using drop and differential scanning calorimetry. Mater. Sci. Eng. A.

[28] Jeya Ganesh B (2010). Differential scanning calorimetry study of diffusional and martensitic phase transformations in some 9 wt-%Cr low carbon ferritic steels. Mater. Sci. Technol..

[29] Ghanei S, Kashefi M, Mazinani M (2013). Eddy current nondestructive evaluation of dual phase steel. Mater. Des..

[30] Zhou L (2014). Quantification of the phase fraction in steel using an electromagnetic sensor. NDT E Int..

[31] Yin W, Peyton AJ, Strangwood M, Davis CL (2007). Exploring the relationship between ferrite fraction and morphology and the electromagnetic properties of steel. J. Mater. Sci..

[32] Sorger Gonçalo L., Oliveira J.P., Inácio Patrick L., Enzinger Norbert, Vilaça Pedro, Miranda R.M., Santos Telmo G. (2019). Non-destructive microstructural analysis by electrical conductivity: Comparison with hardness measurements in different materials. Journal of Materials Science & Technology.

[33] Xu H (2018). Imaging x70 weld cross-section using electromagnetic testing. NDT E Int..

[34] Yu Chang, Jingpin Jiao, Xiucheng Liu, Guanghai Li, Bin Wu, Cunfu He (2018). Measurement of the Hardness of Medium Carbon Steel Using the Magnetic Mixing-Frequency Technique. IEEE Transactions on Magnetics.

[35] Makar J, Tanner B (2002). The *in situ* measurement of the effect of plastic deformation on the magnetic properties of steel. J. Magn. Magn. Mater..

[36] Perevertov O (2017). Influence of the applied elastic tensile and compressive stress on the hysteresis curves of Fe-3%Si non-oriented steel. J. Magn. Magn. Mater..

[37] Qiu F, Ren W, Tian GY, Gao B (2017). Characterization of applied tensile stress using domain wall dynamic behavior of grain-oriented electrical steel. J. Magn. Magn. Mater..

[38] Aghadavoudi-Jolfaei M, Shen J, Smith A, Zhou L, Davis CL (2019). Non-destructive measurement of microstructure and tensile strength in varying thickness commercial DP steel strip using an EM sensor. J. Magn. Magn. Mater..

[39] Perevertov O, Stupakov O, Tomáš I, Skrbek B (2011). Detection of spring steel surface decarburization by magnetic hysteresis measurements. NDT E Int..

[40] Zhu W (2012). Evaluation of rail decarburisation depth using a H-shaped electromagnetic sensor. NDT E Int..

[41] Liu J (2015). Electromagnetic evaluation of the microstructure of Grade 91 tubes/pipes. *Int*. J. Press. Vessel. Pip..

[42] Cedillo E, Ocampo J, Rivera V, Valenzuela R (1980). An apparatus for the measurement of initial magnetic permeability as a function of temperature. J. Phys. E..

[43] Morishita M, Takahashi N, Miyagi D, Nakano M (2011). Examination of magnetic properties of several magnetic materials at high temperature. Prz. Elektrotechniczny (Electrical Rev..

[44] Yamamura H, Toh T, Harada H, Takeuchi E, Ishii T (2001). Optimum magnetic flux density in quality control of casts with level DC magnetic field in continuous casting mold. ISIJ Int..

[45] Kuz’ko EI, Belomyttsev MY, Belov VA (2018). A Study of Phase Transformations in High-Chromium Ferritic-Martensitic Steels by Magnetometry. Met. Sci. Heat Treat..

[46] Harada, H., Nagashima, M., Konno, T., Yamana, M. & Toh, T. Electromagnetic sensor just below CC mold by using magnetic transformation of steel. *IOP Conf. Ser. Mater. Sci. Eng*. **424** (2018).

[47] Sorger, G., Vilaça, P. & Santos, T. G. Concept and Architecture of a New Advanced Control for the FSW of Steels. *Proc. 11th Int. Symp. Frict. Stir Weld*. (2016).

[48] Vilaça Pedro, Thomas Wayne (2011). Friction Stir Welding Technology. Structural Connections for Lightweight Metallic Structures.

[49] Mishra RS, Ma ZY (2005). Friction stir welding and processing. Mater. Sci. Eng. R Reports.

[50] Zhou L, Hall R, Davis CL (2019). Measured and modelled low field relative permeability for dual phase steels at high temperature. J. Magn. Magn. Mater..

[51] Boehm, A. & Hahn, I. Measurement of magnetic properties of steel at high temperatures. *Ind. Electron. Soc. IECON 2014-40th Annu. Conf. IEEE* 715–721 (2014).

[52] Takahashi N, Morishita M, Miyagi D, Nakano M (2011). Examination of magnetic properties of magnetic materials at high temperature using a ring specimen. IEEE Trans. Magn..

[53] Monostori L (2016). Cyber-physical systems in manufacturing. CIRP Ann..

