# Supplementary material for: Local magnetic flux density measurements for temperature control of transient and non-homogeneous processing of steels
Source: Sci Rep. 2019 Nov 29;9:17900. doi: 10.1038/s41598-019-54503-5 (PMC6884644; doi:10.1038/s41598-019-54503-5)
Supplement: Supplementary file 1 — Supplementary Information [file 41598_2019_54503_MOESM1_ESM.docx]

Supplementary Information

Local magnetic flux density measurements for temperature control of transient and non-homogeneous processing of steels

Gonçalo Sorger^1*^, Pedro Vilaça^1^,Telmo G. Santos^2^

^1^ Department of Mechanical Engineering, School of Engineering, Aalto University, 02150 Espoo, Finland

^2^ UNIDEMI, Department of Mechanical and Industrial Engineering, NOVA School of Science and Technology, Universidade NOVA de Lisboa, 2829-516 Caparica, Portugal

# Supplementary Methods

## Material

The base material used in this study was a S700MC high-strength steel produced by TMCP, whose chemical composition is: [max. wt.%] 0.059 C; 0.205 SI; 1.79 Mn; 0.007 P; 0.002 S; 0.026 Al; 0.083 Nb; V; 0.013; 0.113 T. The specimens used in the thermal and thermomechanical processing cycles were 100 x 100 x 4 mm plates. For the thermal cycles (oxy-fuel flame heat source), a region of reduced thickness (2 mm) was produced on the middle of the plates, by removing Ø 25 x 2 mm of material by machining to concentrate the heating effect. Similarly, for the thermomechanical cycles (FSW tool heat source) a Ø 10 mm and 3.5 mm deep hole was drilled at the center of the plates to remove a volume of material roughly equivalent to that of the tool probe.

## Differential scanning calorimetry (DSC)

Differential scanning calorimetry measurements were performed on a NETZSCH STA 449F1 equipment, capable of a maximum heating rate of 50 ºC/s. A sample of the S700MC steel (approximately 1.5 x 2 x 3 mm and 75 mg) was placed inside an Al_2_O_3_ ladle and the measurements were carried out under a protective Argon atmosphere. The thermal cycles comprised four stages: 1 – holding at 100 ºC; 2 – heating (at 50, 20, 10, and 5 ºC/min); 3 – holding at 1000 ºC; and 4 – cooling (at 50, 20, 10, and 5 ºC/min). The cycles were carried out three times per heating/cooling rate. The holding times were 5 min.

## Thermal and thermomechanical cycles with magnetic flux density and temperature measurements

Thermal and thermomechanical cycles were carried out on S700MC HSS using non-electrical heat sources to avoid any coercive electromagnetic interaction with the magnetic measurement system. Supplementary Figure S2 shows the experimental setup. Two heat sources were applied to the specimens: For the thermal cycles it was an oxy-fuel flame, which is a purely thermal heat source; and for the thermomechanical cycles it was the thermomechanical processing, which is an indirect heat source via internal friction dissipation during the plastic deformation induced in the HSS specimen by the plunging and rotation of a rigid FSW tool. The tool material was a non-magnetic polycrystalline cubic boron nitride with ceramic binder. Thermocouples were inserted into small holes reaching the half thickness of the processed zone of the specimens as shown in Supplementary Fig. S2.

The magnet used to generate the magnetic field was a Ø 15 x8 mm NeFeB permanent magnet with N42 magnetization in the axial direction. The distance from the magnet to the test sample was such that the interaction between the field and the sample generates the largest field intensity variation when the material changes from ferromagnetic to paramagnetic (and vice versa) without saturating the signal from the Hall-effect sensors. A good compromise between these two conditions was achieved at a distance of 13 mm, directly under the processed zone. The two sensors used to measure the magnetic flux density were SS496A1 ratiometric Hall-effect sensors. These were positioned in 2 mm deep slots machined on the copper chassis at ± 5 mm from the center of the plate. The positioning of these sensors, relative to the permanent magnet and the test plate, was supported by the results of the magnetostatic computational analysis. The chassis was water cooled to protect the magnet and the magnetic sensors from the effects of the high temperatures. Furthermore, a 3 mm thick thermal barrier (air gap for the thermal cycles and a Ti plate for the thermomechanical cycles) was placed between the copper chassis and the steel test piece for additional protection of the magnet and the sensors from the high temperature, and also to provide additional backing support in the case of thermomechanical cycles. The data from the Hall-effect sensors and the thermocouples was acquired via a NI USB-6008 module and a NI-9212 module, respectively. A custom-made application was created in LabVIEW to control and synchronize the data acquisition and recording.

Supplementary Figure S1 Experimental setup for the magnetic flux density and temperature measurements: (a) The setup used for the thermal cycle with flame as heat source; (b) Details of the measurement system inside the chassis shown for the thermal cycle with flame as heat.


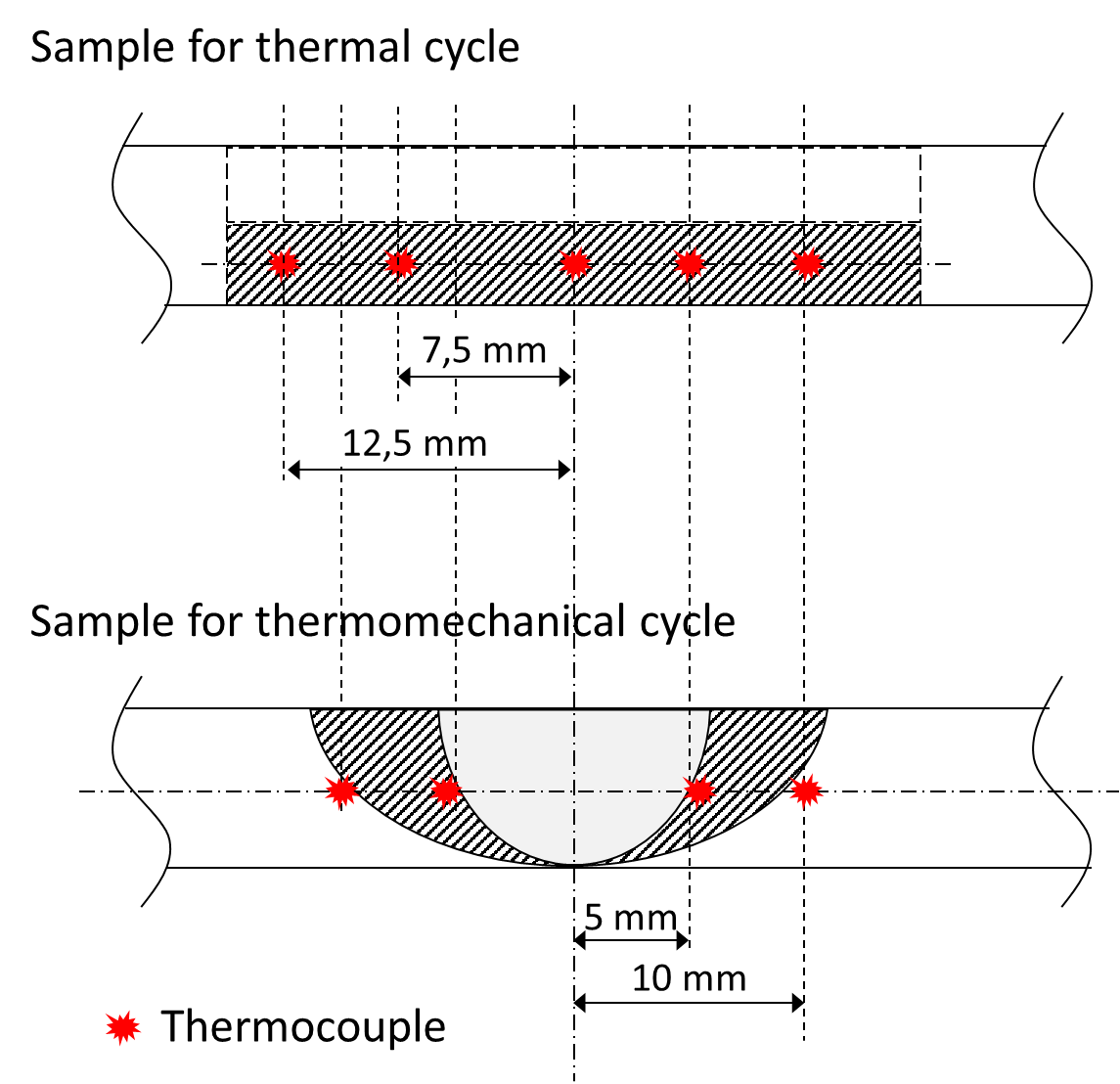


Supplementary Figure S2 Position of the thermocouples at the half-thickness of the specimens.

## Computational analyses

Transient thermal computational analyses were performed using the commercial ANSYS Workbench 19.0 software. The purpose of these analyses was to obtain the temperature fields in agreement with the temperature measurements obtained experimentally via thermocouples and to estimate the volume of material that reached temperatures above T_C_. The geometries used were the same as shown in Figure 1a but with the specific sample geometry for each heat source (i.e. a region of reduced thickness in the case of the flame as heat source, and the negative of the FSW tool in the case of the FSW tool as the heat source). The meshes were comprised of about 3.8 million (flame as heat source case) and 3 million (FSW tool as heat source case) tetrahedron elements. The maximum element size was 0.5 mm in the steel plates and 1 mm in all other bodies. The loading condition representing the flame was a heat flow with a normal distribution applied on the surface of the reduced thickness region of the steel specimen body. The analysis was carried out in one step. The loading conditions representing the effect of the heat flow from the FSW tool were applied on the surfaces corresponding to the negative of the tool geometry. The analysis was carried out in two steps. The first step with heat flow applied only on the surfaces corresponding to the probe, and the second step adding the heat flow contribution on the surfaces corresponding to the shoulder. The final results were obtained by adjusting loading conditions iteratively until the simulated temperature fields were in close agreement with the thermocouple measurements for each case. In both cases, the initial temperature was 25 °C, and a constant temperature of 25 ªC was applied to the surfaces corresponding to the inside of the copper cooling tubes. An emissivity of 0.3 was considered at the top surface of the steel specimen, excluding the heat flow loading surfaces. All other outside surfaces were adiabatic. The material models used were those for Steel 1010, Copper, Titanium, and Air, available in the materials library of the ANSYS Workbench 19.0 software. The results were validated by comparing the temperature fields above the T_C_, obtained computationally, with the heat-affected zones evaluated in cross-sections of samples extracted from the center of the processed specimens. A table with the material thermal properties, figures and graphs supporting the methods implemented in the thermal analyses are included in the “Suplementary Information” available with the online version of this paper.

The magnetostatic computational analyses were performed using the ANSYS Maxwell R18.0 software. The geometries were the same as in the thermal analyses. The meshes were comprised of 2.8 million tetrahedron elements (in both the flame and the FSW tool analyses). The volumes obtained from the thermal analysis were integrated into the magnetostatic model and the analyses were carried out for different values of magnetic permeability (μ_R_ >> 1 vs μ_R_ ≈ 1) in that volume. The material models used were those for Steel 1010, Copper, Titanium, and NdFe35, available in the materials library of the ANSYS Maxwell R18.0 software. A table with the magnetic properties considered for the materials is included in the “Suplementary Information” available with the online version of this paper.

Supplementary Table S1 Thermal properties considered for the materials in the computational thermal analysis.

| Material | Density [kg/m^3^] | Isotropic Thermal Conductivity [W/m.^o^C] | Specific Heat [W/kg.^o^C] |
| --- | --- | --- | --- |
| Steel 1010 | 7872 | 45 | 448 |
| Copper | 8933 | 400 | 385 |
| Titanium | 4500 | 21 | 522 |
| Air | 1.1614 | 0.026 | 1007 |

Supplementary Table S2 Magnetic properties considered for the materials in the computational magnetostatic analysis.

| Material | Relative Permeability | Magnetic Coercivity [A/m] |
| --- | --- | --- |
| Steel 1010 | B-H curve (default form software library) | 0 |
| Copper | Simple (=0.999991) | 0 |
| Titanium | Simple (=1.00018) | 0 |
| NdFe35 | Simple (=1.0997785406) | -890000 (Z direction) |

Supplementary Figure S3 Meshes used in the thermal computational analyses. Cross-section view of the mesh used in the computational analysis of the thermal cycle produced with the flame (a). Top surface perspective of the mesh where the heat flow loading simulating the flame is applied on the body representing the steel specimen (b). Cross-section view of the mesh used in the computational analysis of the thermomechanical cycle produced with the FSW tool (c). Top surface perspective of the mesh where the heat flow loading simulating the FSW tool is applied on the body representing the steel specimen (d). The colors are cosmetic.

Supplementary Table S3 Temperature measurements at different times during the Flame_T_MAX_>T_C_ cycle (Figure 6a in the paper) and the respective magnetic flux density measurements. The temperature measurements were used to validate the computational thermal analysis.

| From experimental measurements | | | | | |
| --- | --- | --- | --- | --- | --- |
| Measurement time [min] | | 2.06 | 2.34 | 2.77 | 4.77 |
| Temperature from Thermocouples | TC1 | 740 | 771 | 808 | 899 |
|  | TC2 | 709 | 740 | 764 | 860 |
|  | TC3 | 672 | 705 | 740 | 837 |
|  | TC4 | 599 | 631 | 667 | 723 |
|  |  |  |  |  |  |
| Magnetic flux density measurements | B | 460.5 | 422.9 | 398.4 | 374.5 |
|  | B_0_ – B_i_ | 39.5 | 77.1 | 101.6 | 125.5 |
| From computational analysis | | | | | |
| Volumes with T_i_>T_C_ | | 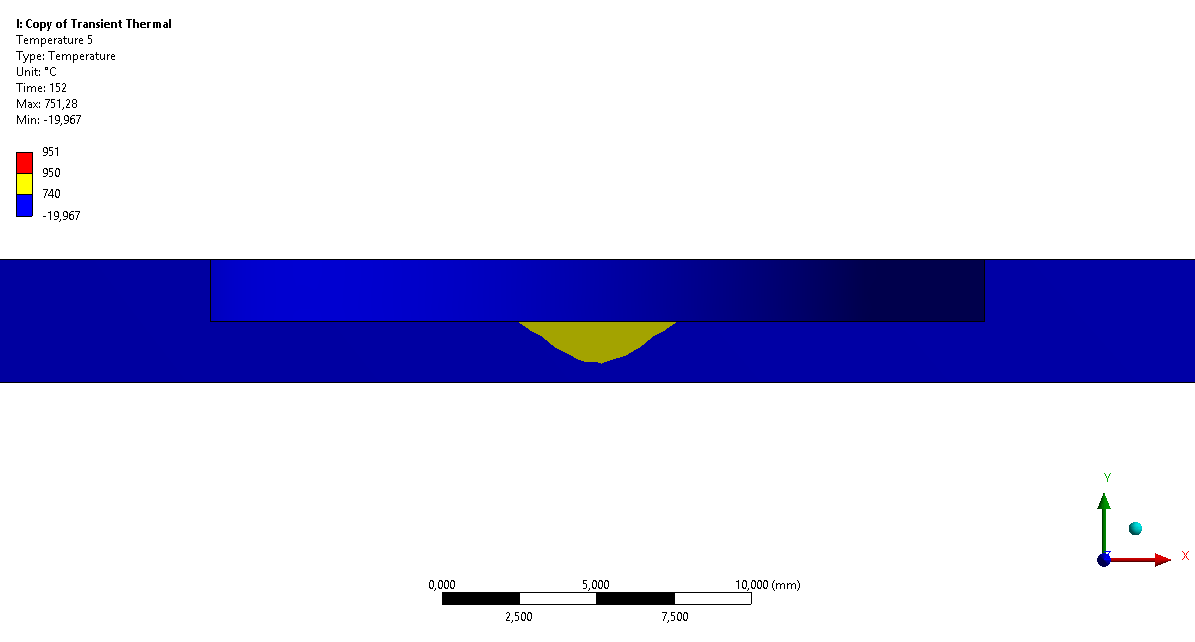 | 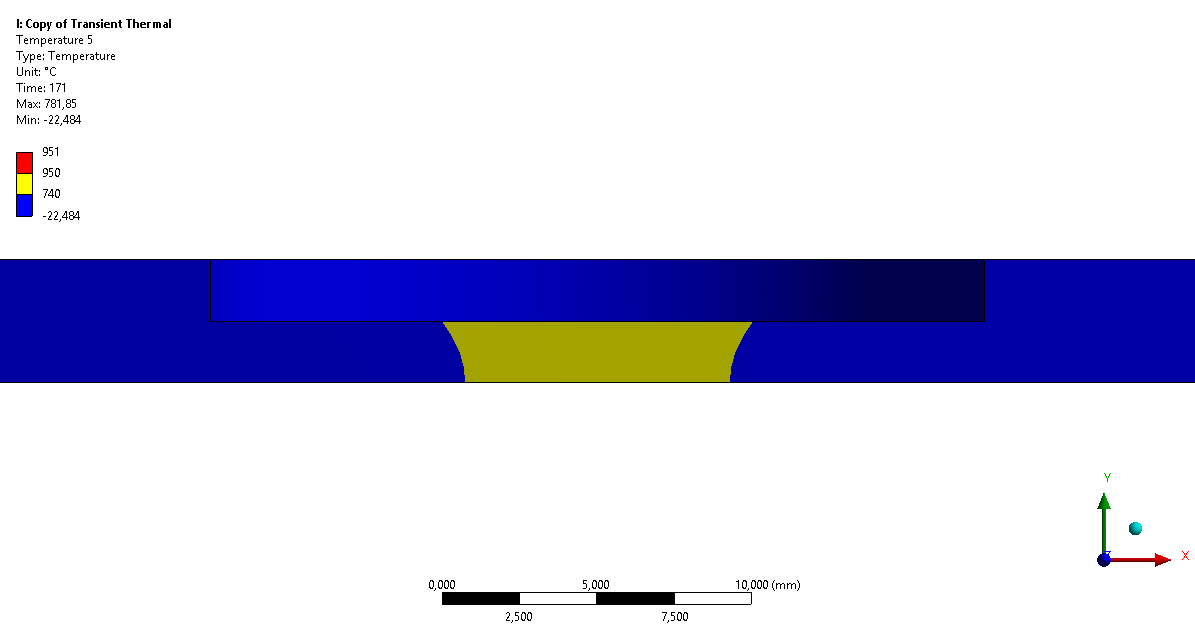 | 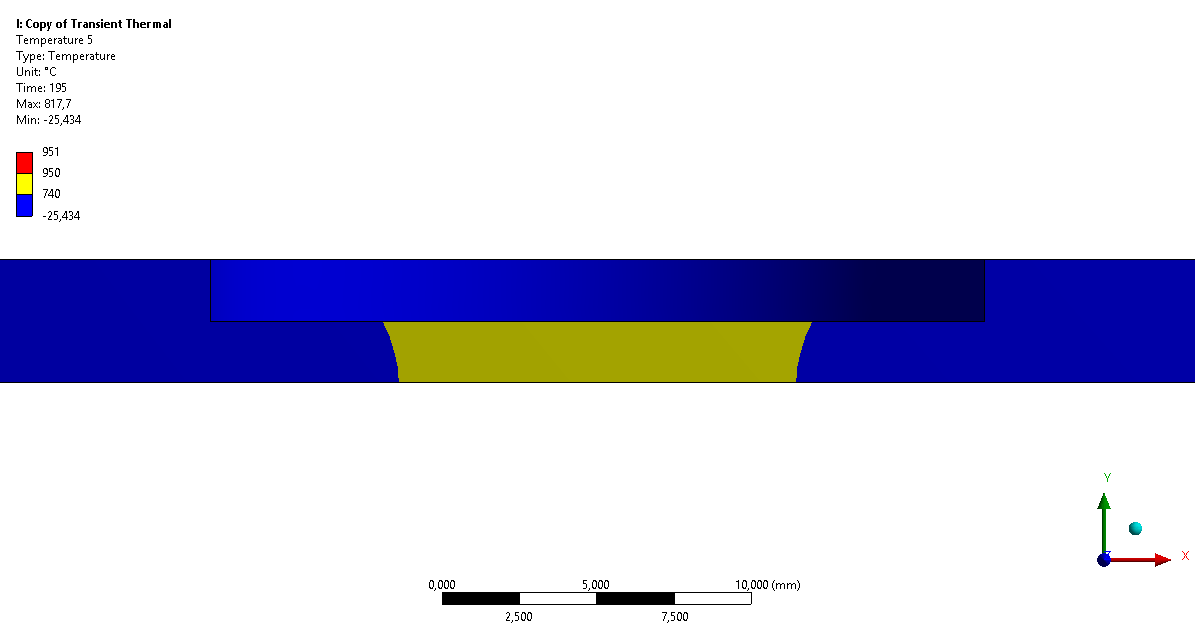 | 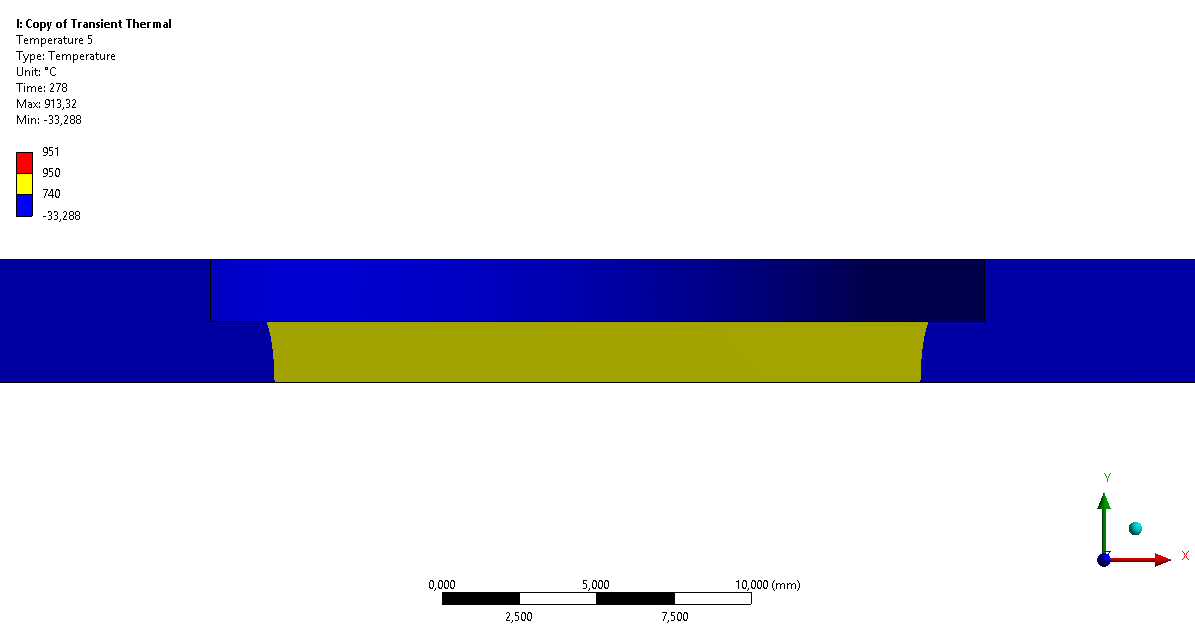 |
| Volume [mm^3^] | | 12 | 131 | 278 | 711 |


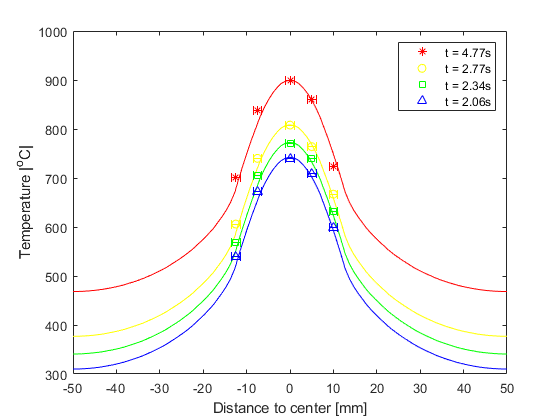


Supplementary Figure S4 Temperature measurements obtained experimentally at different times during the Flame_T_MAX_>T_C_ cycle (Figure 6a in the paper) and temperature distribution curves obtained via the respective computational thermal analysis.

Supplementary Table S4 Temperature measurements at different times during the FSW_T_MAX_>T_C_ cycle (Figure 6b in the paper) and the respective magnetic flux density measurements. The temperature measurements were used to validate the computational thermal analysis.

| From experimental measurements | | | | | |
| --- | --- | --- | --- | --- | --- |
| Measurement time [min] | | 1.97 | 2.23 | 2.29 | 2.99 |
| Temperature from Thermocouples | TC1 | 684 | 783 | 817 | 858 |
|  | TC2 | 611 | 729 | 777 | 812 |
|  | TC3 | 492 | 660 | 732 | 787 |
|  | TC4 | 419 | 574 | 615 | 692 |
|  |  |  |  |  |  |
| Magnetic flux density measurements | B | 490.5 | 454.5 | 443 | 410.5 |
|  | B_0_ – B_i_ | 30 | 66 | 77.5 | 110 |
| From computational analysis | | | | | |
| Volumes with T_i_>T_C_ | | 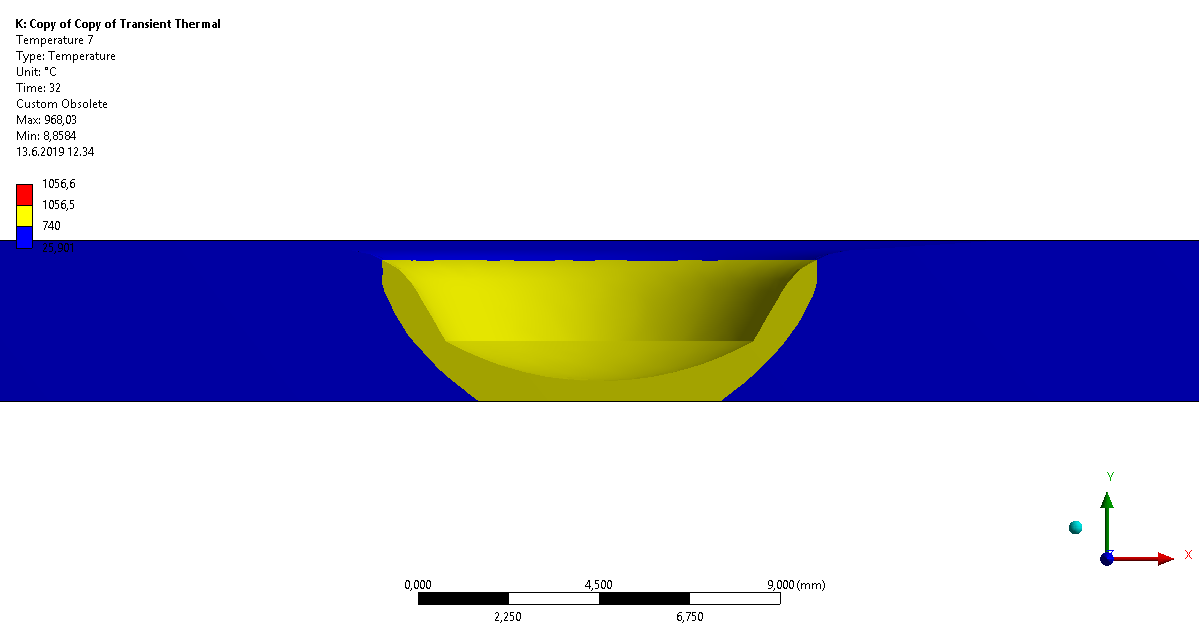 | 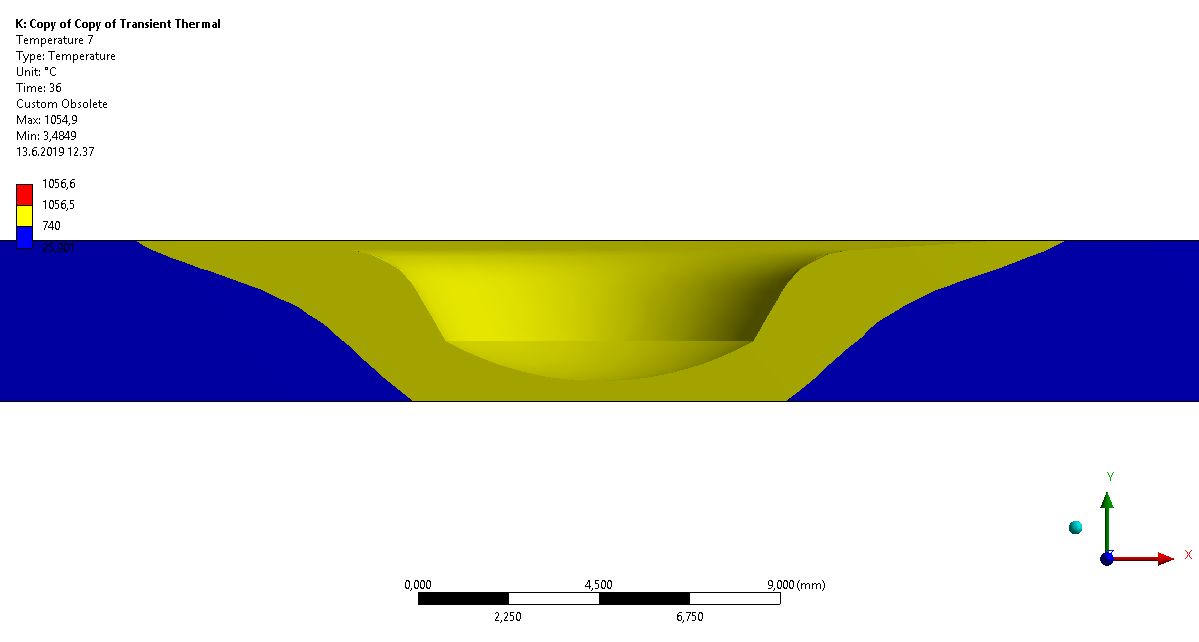 | 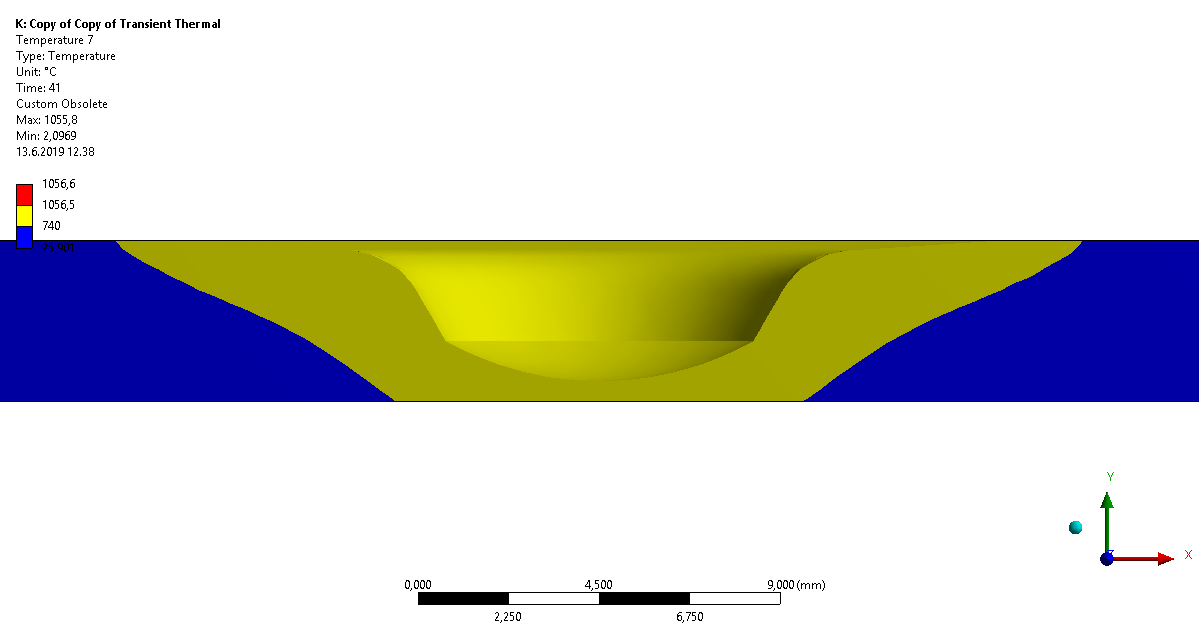 | 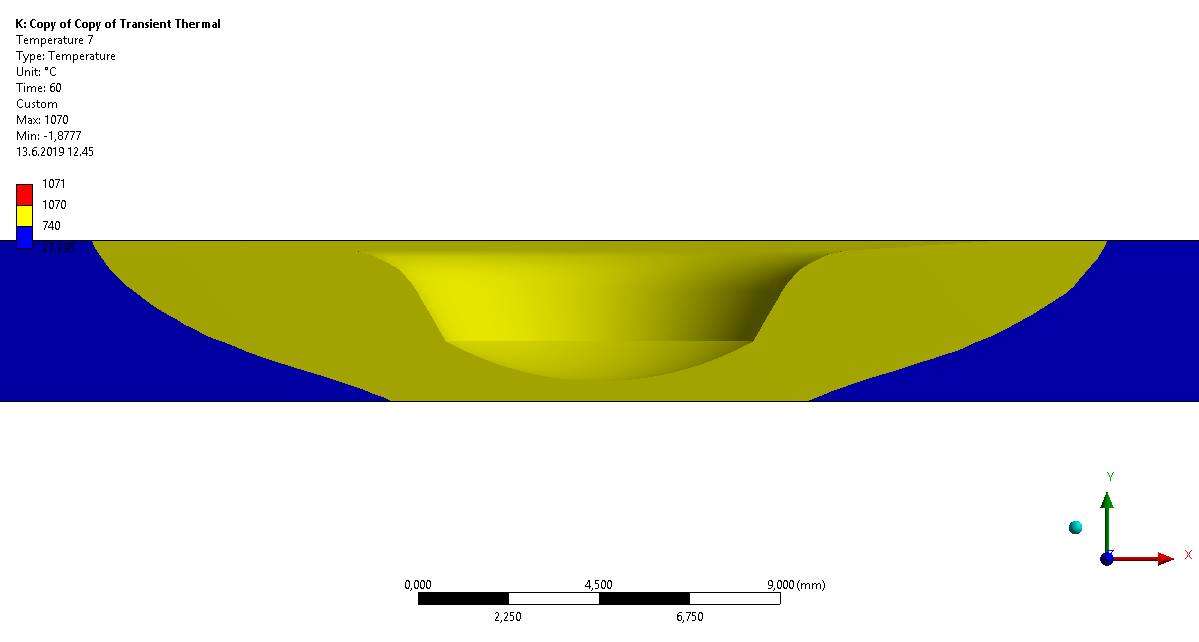 |
| Volume [mm^3^] | | 95 | 503 | 723 | 1106 |


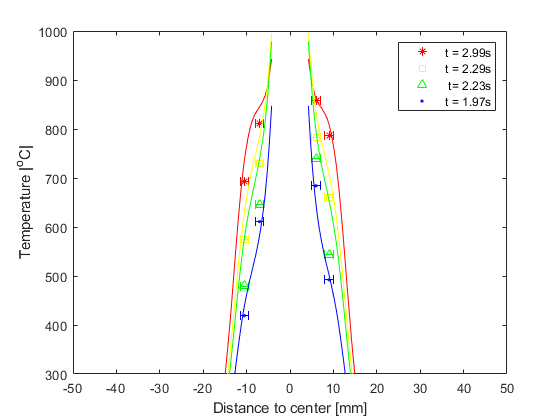


Supplementary Figure S5 Temperature measurements obtained experimentally at different times during the FSW_T_MAX_>T_C_ cycle (Figure 6b in the paper) and temperature distribution curves obtained via the respective computational thermal analysis.
